# On the purpose of meaningful human control of AI

**DOI:** 10.3389/fdata.2022.1017677

**Published:** 2023-01-09

**Authors:** Jovana Davidovic

**Affiliations:** ^1^Department of Philosophy, The University of Iowa, Iowa City, IA, United States; ^2^Stockdale Center for Ethical Leadership, United States Naval Academy, Annapolis, MD, United States

**Keywords:** machine learning and AI, war, meaningful human control, ethics, robots

## Abstract

Meaningful human control over AI is exalted as a key tool for assuring safety, dignity, and responsibility for AI and automated decision-systems. It is a central topic especially in fields that deal with the use of AI for decisions that could cause significant harm, like AI-enabled weapons systems. This paper argues that discussions regarding meaningful human control commonly fail to identify the purpose behind the call for meaningful human control and that stating that purpose is a necessary step in deciding how best to institutionalize meaningful human control over AI. The paper identifies 5 common purposes for human control and sketches how different purpose translate into different institutional design.

## Introduction

All around us algorithms are making decisions about us and for us. From how we chose what to watch, to how we shop, get policed and go to war, get social services, medical diagnoses, or loans, algorithms are quite literally everywhere. No aspect of our lives is unaffected by algorithms whose incredible power promises to continue to change our lives. AI, big data, and machine learning will help us address climate change, cure cancer, feed more people, and fight less bloody wars. But with this incredible power comes great potential for harm. In fact, the very features that make AI a powerful tool also make it very dangerous. These features include the ability to process large data sets that humans cannot, the ability to “see” patterns humans could not, the ability to apply solutions on grand scales, and the ability to do so at great speed. These abilities and their driving force—machine learning—make AI not only capable of causing harm, but also less transparent, less explainable, and often unfair and unjust. Much has been said about these issues (Mittelstadt et al., 2016; Felzmann and Villaronga, 2019; Larsson and Heintz, 2020; Brown et al., 2021). Lawyers, scholars, and the public have, for example, repeatedly called for transparency and explainability, arguing that we cannot leave morally consequential decisions to machines. Instead, they argue, we need human-machine teams, and the necessary transparency and explainability for those teams to work.

Most importantly, many scholars argue, we need meaningful human control over these powerful algorithms.^1^ After all, we need a human to stop a drone attack on a person identified by a classification algorithm as a combatant, but carrying a carpet rather than a rocket launcher. We need a human to question or re-assess a recidivism risk assessment when the algorithm is known to be racially biased and intentionally designed to err toward false positives. We want a human to question the predictions of a climate model not trained on the data relevant to geographic location within which we are trying to apply it. Simply put, we need to make sure that the algorithms we use do not cause more harm than they can spare us and to do so we need meaningful human control (UNIDIR, 2014). For example, the U.S. military has called for meaningful human control over certain systems or what they refer to in DoD directive 3,000.09—“appropriate human judgement” (Department of Defense, 2016). Similarly, the U.N. Committee for the Convention on Certain Conventional Weapons (CCW) has argued that in spite of the fact that “there is not yet an internationally agreed definition of what precisely meaningful human control constitutes, there is…convergence that some degree of human control over…LAWS is vital” (Schwartz, 2018). The European Commission also recently proposed a regulation that stipulates that ““high-risk A.I. systems” (such as facial recognition and algorithms that determine eligibility for public benefits) should be designed to allow for oversight by humans who will be tasked with preventing or minimizing risks” (Green and Kak, 2021). The GDPR (European General Data Protection Regulation) also assures the right not to be subjected to a decision based on solely automated process (European Union Parliament, 2016). Examples go on, but the main takeaway is that the primary effort to mitigate risks of harm from ADS (automated decision-systems) and AI across jurisdictions focuses on assuring “meaningful human control.” The problems with this approach are many—from the fact that scholars do not agree on what meaningful human control is, to automation bias, i.e., the tendency to trust machines when machines and humans opinions conflict, to the worries that we cannot expect humans to meaningfully provide oversight for the very systems that were built because of and for things humans do not have the capacity to do (Green and Kak, 2021). These are significant problems for sure, and solving them will be key for mitigating the risks of ADS and machine-learning AI. But here I want to focus on what I see as the *conceptually primary problem*—clarifying the purpose of meaningful human control.

The success of meaningful human control as a “solution” for the woes of AI depends on the problem one is trying to solve for. Generic calls for meaningful human control are unhelpful and have consistently led to generic descriptions of what meaningful human control would look like. Discussions of “meaningful human control” most often focus on who should and when exert control over the process, without ever explicitly asking, for which purpose or why (Roff and Moyes, 2016; Ekelhof, 2018). Simply put, meaningful human control can solve different problems and serve different purposes and as such it requires different institutional design for different aims. Thus, the first step in deciding whether we need meaningful human control, and what shape that human control ought to have, as well as what do we need to successfully exert such control (e.g., what type of explanations or information) depends on the purpose of that control. In what follows, I lay out the 5 main purposes that human control of automated decision systems could serve, and then I concisely explain why and how different purposes require different institutional design and types of different explanations of ADS outputs.

## Purpose axis—The five purposes of meaningful human control

a. Safety and precision: One, common, reason for human control over AI systems is accuracy, safety, and precision. In many cases, the reason we hope to have a human in the loop is because we think that that will prevent mistakes and avoid harm. Such calls for “humans in the loop” make sense in cases when humans are better at some cognitive task (object recognition—for now), or when context affects outcomes and is difficult to model, or in cases when unanticipated changes to our environments might occur. In cases when a human together with a machine performs better than a machine alone, safety and precision are an obvious reason to have meaningful human control. Of course, such control might not be possible in cases where large sets of data are processed by the algorithm or when the speed of processing or the need for speed of decision-making is what makes the AI particularly valuable (e.g., anti-missile or anti-drone swarm ship defense systems). Centrally, when the aim of human control is safety, the location of the human in the loop in the decision-making chain, should obviously be driven by increase in safety and precision. Whether the human should be the final decider, or just an oversee-er, or only have control over deployment more generally, when safety is primary concern, should be solely driven by empirical analysis- whatever works more effectively.
b. Responsibility and accountability: Sometimes, meaningful human control is, however, primarily meant to assure accountability and responsibility. In as much as machine learning algorithms or semi-autonomous or autonomous AI play a role in decisions that might lead to lethal harm or other types of significant harm, institutions using such AI, might be interested in knowing who to hold responsible for potential failures and resultant harm. Where responsibility chains are already prescribed, one might be interested in knowing how to adjust those responsibility chains in cases when a decision relies on AI. We might, for example, ask how to distribute responsibility between developers, acquisition teams, and those that choose to deploy the system in a particular setting. If our primary concern is assigning responsibility, we might “insert” a human in a different part of the algorithms' life cycle then we would have if our primary concern is safety. For example, unlike the cases when our primary concern is safety, in cases where we want meaningful human control for purposes of responsibility, we might take into consideration previous responsibility assignments, or even arbitrary assignments of human control (as long as they are clear).
  It is worth nothing that responsibility assignment and accountability might not require the same solutions and are not identical. Accountability, in some cases, simply requires that we know why the decision was A rather than B (for example so we can assess whether the reasons used for a decision were constitutional, or fair, or reasonable). Accountability might, therefore, at times, be satisfied by a simple technological solution. For example, a meta-interpretive algorithm like LIME (Local Interpretable Model Agnostic Interpretations) (Ribeiro, 2016). Responsibility assignments cannot, in contrast, be satisfied technologically. Responsibility, at least for now, requires a human in the loop for different reasons—because as it stands we can't hold machines responsible in any meaningful sense.
  In addition to the fact that the shape and location of human control for purposes of accountability and for purposes of responsibility vary, it is also important to note that there is a range of types of responsibility-purposes. For example, assignments of moral responsibility and assignments of legal responsibility might require different types of institutional design for “meaningful” control. When assignments of responsibility are the reason behind the calls for meaningful human control, it matters greatly whether we are after:

b. i. Legal responsibility.
  1. Forward-looking (for which corporate liability models might act as a potential model) (Elish, 2019; Selbst, 2020; Diamantis, 2021).
  2. Backward-looking (for retributive or restorative justice).

b. ii. Moral responsibility.
  1. Moral responsibility for assigning blameworthiness.
  2. Moral responsibility for assigning liability to defensive harm.
  3. Moral responsibility for assigning liability to punitive harm.

c. Morality and dignity: Another common reason people have called for meaningful human oversight is to solve for problems they see with harm and especially lethal harm being imposed by fully autonomous weapons systems, sometimes called “killer robots” (Horowitz and Scharre, 2016). Those arguing against killer robots usually argue that fully autonomous AI doesn't have key moral features (moral reasoning for example) and thus meaningful human control is needed to justify lethal harm (Purves et al., 2015). Others argue that to be killed by a machine violates human dignity and thus a human is needed in the loop any time lethal harm is considered. Meaningful human control for purposes of assuring dignity of targets will obviously take a very different form than meaningful human control for purposes of, for example, legal responsibility. For example, while legal responsibility can be captured by some kind of strict liability approach—in which case owner of the ADS would be the one considered in “control” and thus responsible for its malfunction, dignity on most accounts requires that a human is the final link in the kill chain, and in a meaningful sense—the “proximate cause” of one's death.
  Of course, issues of this kind also exist outside of warfighting contexts—there might be dignity-related reasons to want human control over, for example, biomedical decisions—like end of life decisions, or over the distribution of medical resources, or social services. One might argue for example that there is something morally problematic with leaving medical decisions to ADS without a human in the loop even when safety and precision are not at stake.
d. Democratic engagement AND consent: Often, human control and engagement, have little to do with, or are only instrumentally related to, lowering harm and increasing safety, but instead, are required for procedural justice and fairness. Sometimes we might want stakeholders or those to whom the algorithm is applied to, to have sufficient understanding of the process to consent or dissent and in that way provide human oversight and control over the algorithm (Brennan-Marquez and Henderson, 2019; Pasquale, 2021). In these cases, the benefit of the control is primarily aimed at either democratic engagement or justified consent, and in these cases, the institutional shape that meaningful human control will take will obviously be quite different from cases where it is meant to simply or solely minimize harm. For example, we might be interested in human control over parole decisions, not only to have a recidivism risk tool that is precise and has equal and small false positive rates across racial groups, but we might also want enough transparency in such algorithms so that those to whom the algorithm is applied can challenge specific assessments/outputs of the algorithms as they apply to them. Similar arguments can be given for transparency and explainability for any juridical ADS—namely that one shape meaningful human control can take is ability to question the decisions by such ADS.
e. Instituional stability: There might also be times when the benefit of meaningful human control is really only in the appearance of such control- this might have to do with cases when we want to provide reasons for trust in the institution (Brennan-Marquez et al., 2019). If we are solely after the appearance of meaningful human control, such “control” might look very different, then if we are after the control for one of the above reasons. There might for example be times where appearance of meaningful human control is simply the best we can do, but as a matter of institutional success and stability, such appearance of human control is helpful. Arguably, some autonomous vehicle systems might still rely on having a human on the loop (as a back-up) even if and when that doesn't statistically alter safety, if it increases the trust of pedestrians and society. Whether or not these are good reasons to have meaningful human control, is less relevant here, what matters is that when this is the (or a) reason for such control, it should drive the institutional design around “meaningful control.”

It should be noted that more than one of the purposes discussed here could be behind any particular call for meaningful human control, but being explicit about the main purposes and understanding the institutional design that would best serve each purpose is a crucial first step in trying to make the changes so many are calling for.

Let me finally say a bit more about what it means to say that knowing the purpose of meaningful human control drives institutional design. Understanding the purpose behind calls for “meaningful human control” will provide: (a) the building blocks for the type of explanations we might need and (b) the appropriate location for the meaningful human control. In fact this is the primary reason we should care about carefully and explicitly stating the purpose behind a call for human control over some automated decision system.

Regarding explainability, explainable AI is needed, scholars argue, to be able to exert meaningful control, to be able to justify our actions to citizens, and to be able to question and challenge an ADS decision (Alan Turing Institute, 2020). Scholars often follow up calls for explainable AI (XAI), by lamenting that fully explainable AI is not possible and thus we are stuck with all the problems or many of the problems of ADS (Newman, 2021). But it matters greatly what kind of explanations we are after. We do not always need full explainability, and type of control we are after drives the type of explanation we are after. There is an abundance of literature on explainable AI and many techniques are being developed to apply to (for example classification) algorithms. Developers are opting for more explainable methods more often. Knowing why we need human control and at which stage drives the shape we want XAI to take in a particular setting. Explicit statement of purpose of meaningful human control will thus not only help shape institutional design around the algorithm, but also the shape our explanations need to take to satisfy that purpose. And thus, knowing the purpose of human control, will allow us to be more precise in asking for explainable AI. For example, for some end users whose primary focus in safety it is sufficient that they know common ways a system might fail- and really the only “explanation” they need is to know when not to trust the system. Others who might need to exert control over an algorithm might need to be provided explanations regarding training data- so as to be able to anticipate when a system might not perform well in a new environment. In cases when our primary reason for a call for meaningful human control is responsibility assignment, we probably want the person responsible to have enough of an explanation to be able to form a justified belief—otherwise they may never justifiably use an algorithm and on some accounts of responsibility might never be responsible for negative outcomes, since they wouldn't be held responsible for their ignorance.

Similarly, knowing and explicitly stating the purpose of human control will drive the location where such control is best exerted. If we think of an ADS system's life-cycle, it includes development and design, procurement, deployment within a particular context, and the effects on downstream stakeholders. As we have seen from examples above what meaningful human control looks like and where it is best situated will depend on its purpose. Broadly speaking, for democratic engagement it will have at least a component in affected stakeholders, and for safety and reducing harm it better be situated in the deployment step, while for responsibility assignments, we might have more freedom how we distribute meaningful human control.

Meaningful human control is not a single solution for a single problem, but a tool for a variety of often unrelated problems that arise when using machine-learning AI and automated decision systems. The purpose of human control of AI should be explicitly stated and should drive institutional design. When the purpose of human control is clearly stated it can also provide guidance regarding the kinds of explainability that might be needed in a particular setting.

## Data availability statement

The original contributions presented in the study are included in the article/supplementary material, further inquiries can be directed to the corresponding author.

## Author contributions

The author confirms being the sole contributor of this work and has approved it for publication.

## Footnotes

^1^What we mean by “meaningful human control” is an open question. In the narrowest of senses, it means having a person who presses a button somewhere during each use of the AI in question. In the broadest of senses, it means oversight of processes-either in use of AI or in production, acquisition and use of AI.
